# Critical Considerations in Emergency Repair of Giant Bilateral Inguinoscrotal Hernias

**DOI:** 10.7759/cureus.80242

**Published:** 2025-03-08

**Authors:** Panuwat Pornkul, Renae Bertucci, Kate Swift, Chrispen Mushaya

**Affiliations:** 1 Department of General Surgery, Townsville University Hospital, Townsville, AUS; 2 Department of General Surgery, Cairns Base Hospital, Cairns, AUS; 3 Department of Colorectal Surgery, Townsville University Hospital, Townsville, AUS

**Keywords:** acute abdomen in hernia, giant hernia, giant inguino-scrotal hernia, giant prosthetic reinforcement of the visceral sac, rives-stoppa, trans-abdominal pre-peritoneal mesh repair

## Abstract

This article examines the emergency surgical management of giant bilateral inguinoscrotal hernias through a representative case report. In this case, surgical intervention was required due to an associated cecal volvulus. While acute complications such as bowel strangulation are rare in hernias of this size, delayed presentation and the lack of timely surgical consultation permit significant inguinoscrotal hernia growth. Furthermore, underlying medical comorbidities in these patients may preclude the safe administration of general anesthesia, further hindering timely surgical repair.

In developed countries, both trainees and consultants often have limited exposure to such cases, resulting in minimal experience managing the technical challenges involved. Key factors for successful surgical repair include addressing the loss of domain and managing extensive bilateral anatomical defects. This case report outlines the acute management of a cecal volvulus and the use of an open Rives-Stoppa repair, a technique commonly employed for recurrent, large, or complex inguinal and ventral hernias. By detailing our surgical approach and relevant surgical considerations, this case report provides an educational opportunity to understand the critical challenges of managing giant inguinoscrotal hernias.

## Introduction

This article presents a case of emergency repair for chronic, giant bilateral inguinoscrotal hernias complicated by cecal volvulus, leading to acute scrotal pain and obstructive symptoms. Giant inguinoscrotal hernias, defined as extending beyond the midpoint of the inner thigh while standing, can reach significant size due to patient neglect or delayed surgical repair [1]. Consequently, they are more prevalent in developing countries with socioeconomic barriers to healthcare access [2].

While giant inguinoscrotal hernias are more prevalent in developing countries, they also occur in developed nations like Australia. This is often observed in specific patient populations within Australia, such as those with significant medical comorbidities that preclude safe general anesthesia, a typical requirement for surgical repair of large, complex hernias. Underlying cardiorespiratory issues can also be exacerbated by the sudden increase in intra-abdominal pressure (IAP) after hernia surgery [2]. Consequently, in these high-risk patients, the risks of surgical repair may outweigh its potential symptomatic benefits. Comorbid patients may also be more susceptible to wound-related infections or other perioperative complications; consequently, non-surgical management may be more appropriate for these patients. Although acute surgical complications like bowel strangulation are rare in large inguinoscrotal hernias due to their wide-necked defects, their occurrence creates a complex surgical scenario, exacerbated by the relative infrequency of these cases in Australian clinical practice.

This case report illustrates the effectiveness of the Rives-Stoppa repair technique for complicated giant inguinoscrotal hernias. This method achieves tension-free reinforcement with pre-peritoneal mesh; this method minimizes the risk of recurrence and facilitates the restoration of the abdominal domain [3].

## Case presentation

**Figure 1:**
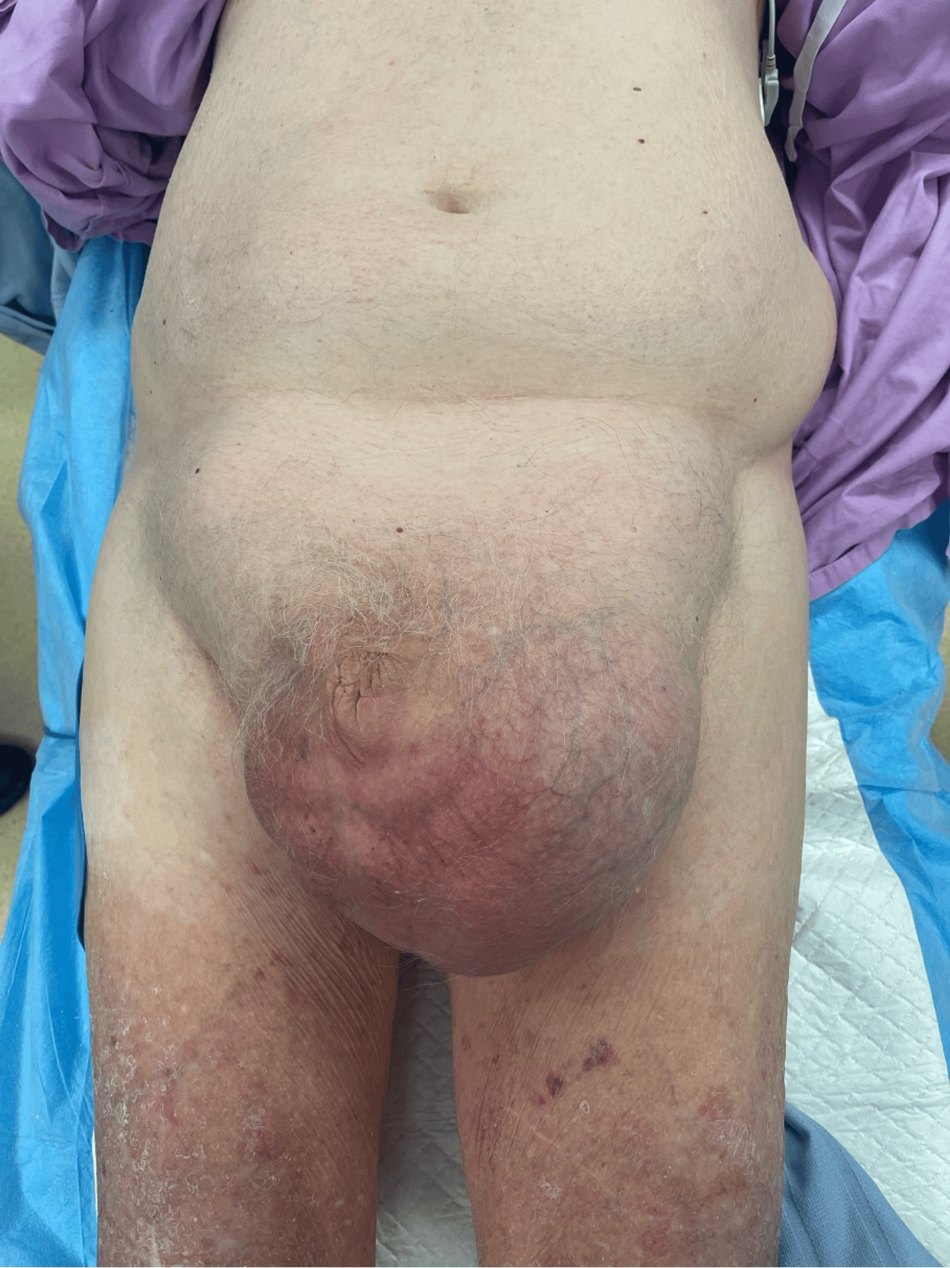
Preoperative clinical image with the patient positioned supine.

An 87-year-old man presented with an eight-hour history of acute severe right scrotal pain and vomiting, without associated abdominal pain. His bowels had last opened the day prior to symptom onset. He was already known to the general surgical department for longstanding, minimally symptomatic giant bilateral inguinoscrotal hernias (Figure 1) and had previously been deemed unfit for surgery following assessment by both a board-certified general surgeon and an anesthetic consultant.

His medical history included severe chronic obstructive pulmonary disease (COPD) with forced expiratory volume in one second (FEV1) of 31% predicted, chronic kidney disease, cutaneous squamous cell carcinoma requiring radiotherapy, hypertension, and gastroesophageal reflux. He was an ex-heavy smoker with a history of alcoholism but did not have cirrhosis. Residing in a retirement village with assisted living support, he was independently mobile but exhibited poor exercise tolerance, limited to 10 meters.

**Figure 2:**
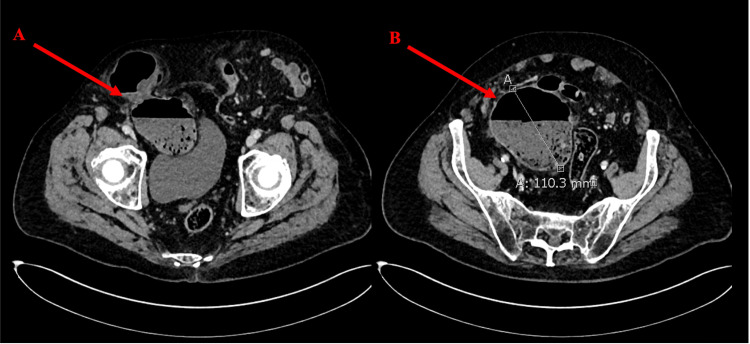
Contrast-enhanced computed tomography axial view of the pelvis. Large bilateral inguinal hernias are demonstrated. Label A demonstrates herniation of the distal descending colon and cecum into the hernia sac through a pinching direct defect. Label B demonstrates the cecum dilated to 11 cm with associated fecalization and a small amount of surrounding free fluid.

On admission, inflammatory markers were normal, and his lactate level was 1.4 mmol/L. The hernias were bilaterally irreducible, with the right side acutely erythematous and tender. Contrast-enhanced abdominal computed tomography (CT) revealed sections of poorly enhancing cecum within the right-sided hernia and mild amounts of associated free fluid, which was clinically concerning for strangulation (Figure 2). On the basis of suspected bowel compromise, the patient was offered emergency surgery after providing informed consent, with a clear acknowledgment that significant risks were associated with the procedure; these were primarily related to his poor preoperative comorbidities.

**Figure 3:**
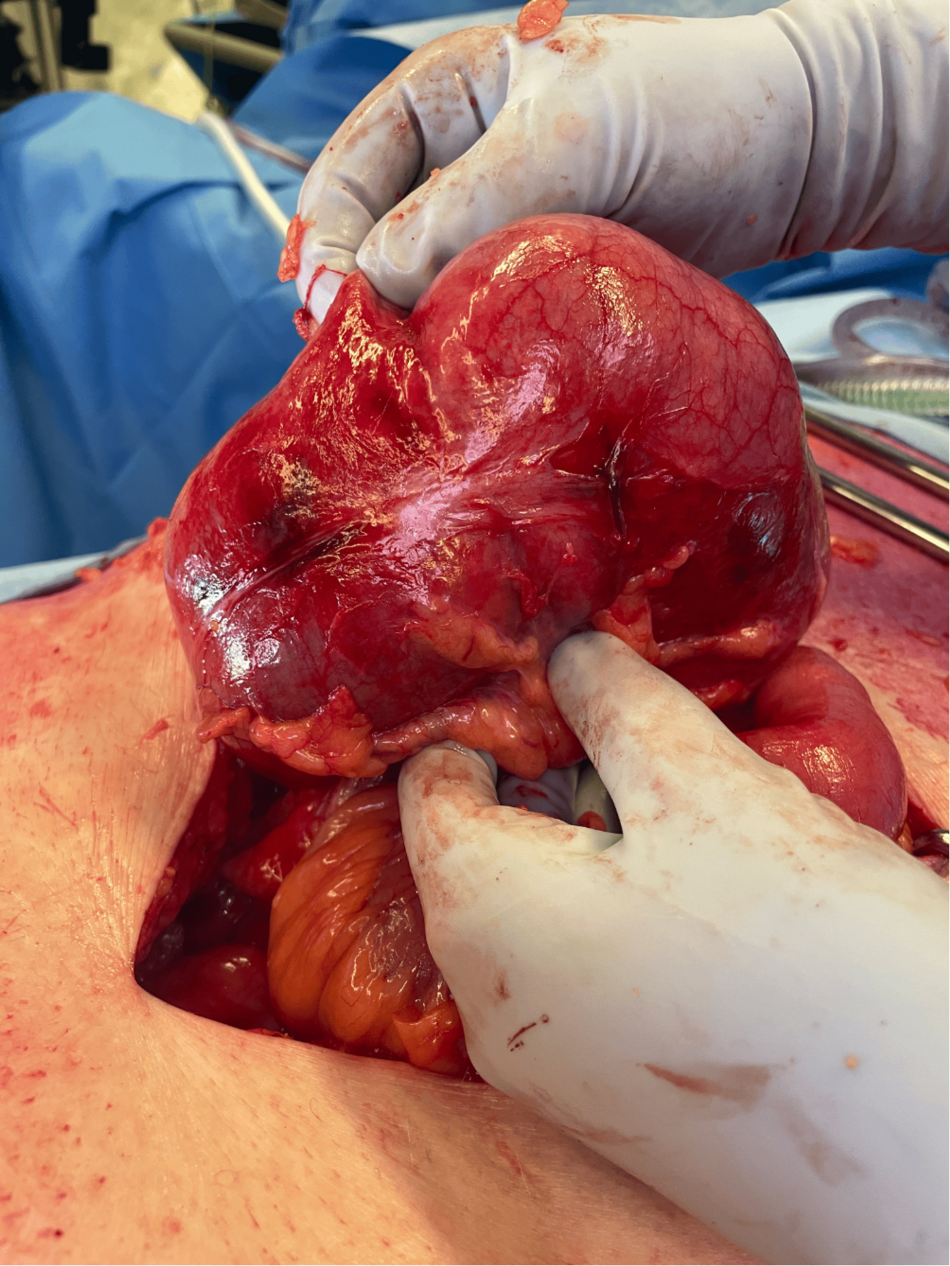
Compromised cecum demonstrating a pattern of patchy ischemia found within the right-sided inguinal hernia sac.

Emergency surgery was arranged, and the patient was positioned supine with nasogastric and urinary catheters in place. A midline laparotomy was performed, which identified a cecal volvulus within the right-sided hernia with evidence of patchy ischemia (Figure 3). These findings necessitated a standard right hemicolectomy with stapled end-to-side ileocolic anastomosis in the absence of perforation or gross contamination of the peritoneal cavity. Furthermore, the patient's cardiorespiratory status remained stable throughout the initial procedure. Given these favorable factors and the inherent benefits of mesh reinforcement in preventing recurrence, the decision was made to proceed with bilateral Rives-Stoppa inguinal hernia repair with mesh. A complete omentectomy was performed to rectify the loss of domain.

The use of mesh in hernia repair after bowel resection, particularly in ischemic bowel cases, requires careful consideration. While mesh is generally avoided in contaminated fields due to infection risk, this case posed unique challenges. Given the patient's increased anesthetic risk and the need to prevent future major surgery, a definitive hernia repair to minimize recurrence was deemed essential.

**Figure 4:**
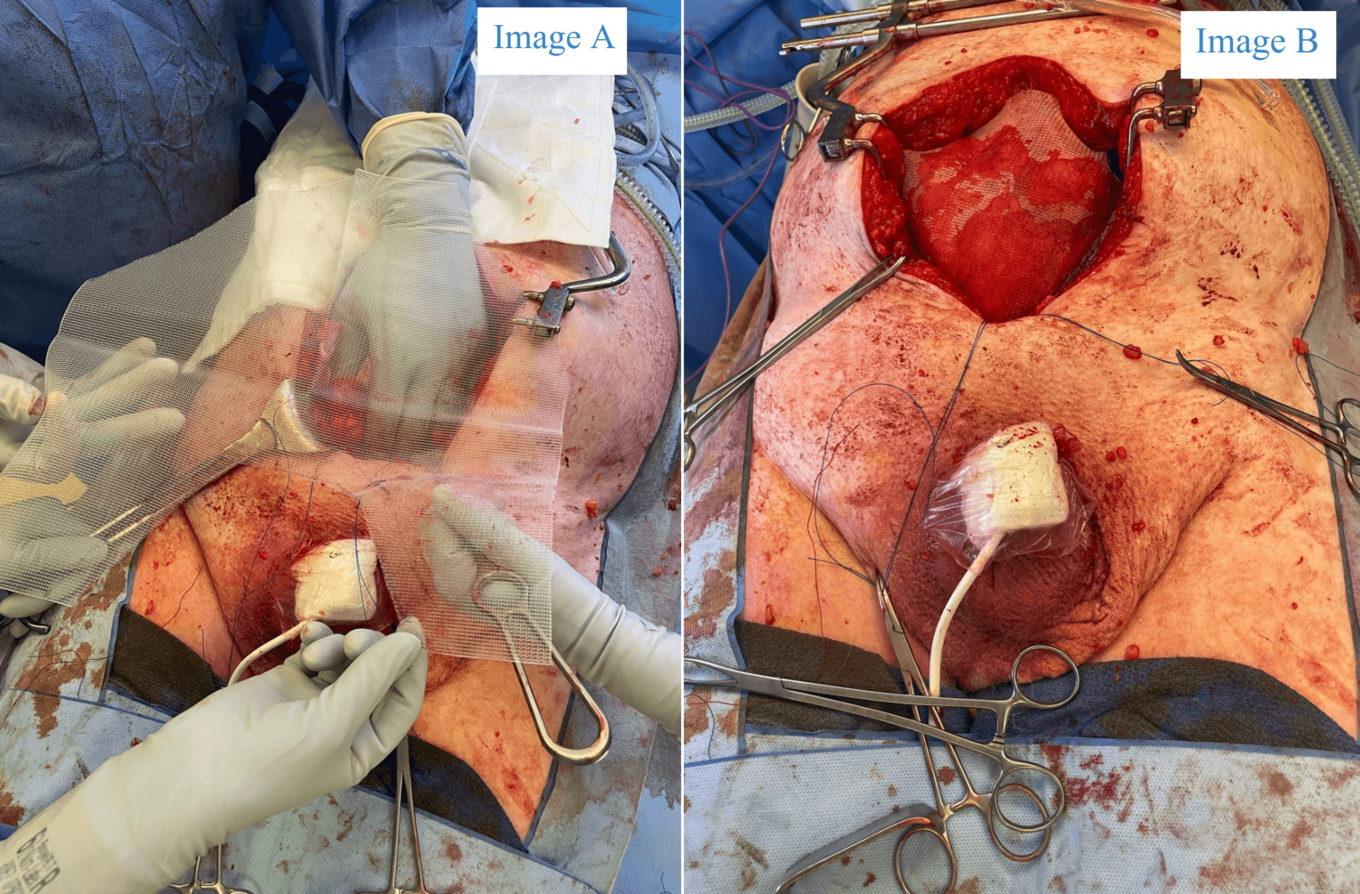
Giant prosthetic reinforcement of visceral sac (GPRVS) as per the Rives-Stoppa technique. Image A (left) demonstrates 30 x 30 cm Prolene mesh cut into a chevron shape and intended positioning. Image B (right) demonstrates the final mesh placement within the retrorectus and pre-fascial plane prior to the closure of the overlying anterior rectus sheath.

The preperitoneal space, including the retropubic space of Retzius and the retroinguinal space of Bogros, was dissected to create a pre-peritoneal space providing surgical access to the bilateral myopectineal orifices; the retrorectus space was also defined superiorly. The remaining hernia contents were reduced back into the abdominal cavity, and orchidectomy was not indicated as the testes were not compromised. The cord structures were parietalized to achieve a tension-free repair; the peritoneal defect related to the hernia was closed with 3/0 Vicryl, and the posterior rectus sheath was closed with 1/0 Polydioxanone (PDS) sutures. The inguinoscrotal hernias were reinforced with a 30 x 30 cm (width x length) Prolene mesh, shaped in a chevron configuration, for giant prosthetic reinforcement of the visceral sac (GPRVS) per the Stoppa technique. The mesh covers the myopectineal orifices and the lower anterior abdominal wall; it was secured to the pubic tubercle and posterior rectus sheath with Tisseel. Two 19 Fr drains were placed in the retropubic space (Figure 4). The anterior rectus sheath was closed over the inserted mesh with 1/0 PDS sutures, and the wound was irrigated, stapled, and covered with a Prevena™ wound therapy device (3M, USA). Rectus sheath catheters were inserted by the anesthetic team.

**Figure 5:**
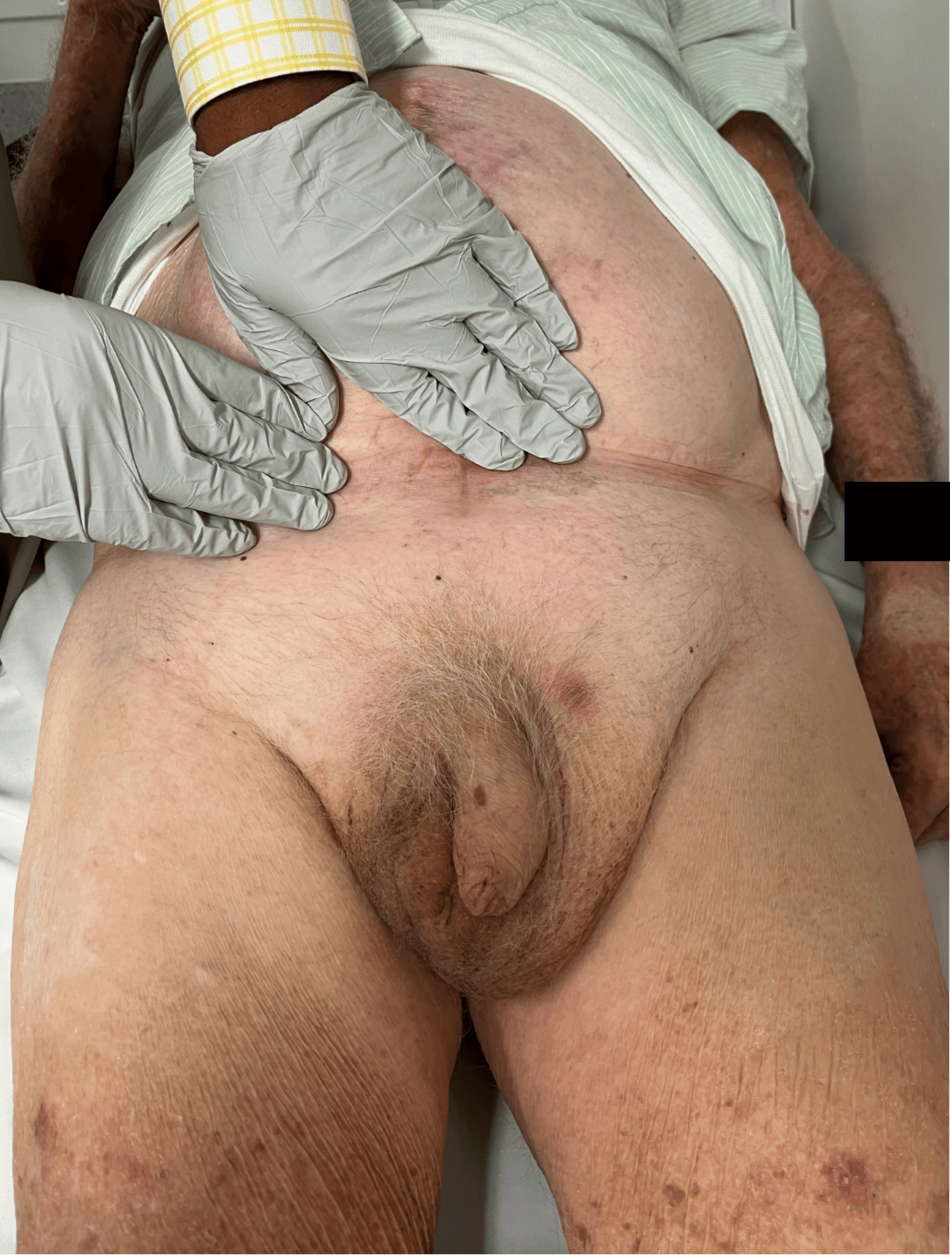
Postoperative image demonstrating satisfactory progress at three months.

The patient was subsequently transferred to the intensive care unit for postoperative care. He was extubated that evening and weaned off inotropic support within 48 hours, transitioning to the ward on postoperative day 3. Pain management included oxycodone patient-controlled analgesia (PCA) and rectus sheath catheters for four days. Drains were removed on postoperative days 1 and 3, respectively. Early enteral intake was initiated, supplemented with parenteral nutrition, and his bowels opened on postoperative day 5. Intensive chest physiotherapy was provided throughout recovery. Postoperative complications included atrial flutter and a superficial wound infection without mesh involvement, managed with electrolyte replacement and antibiotics. The patient was discharged on postoperative day 26 to his original residence at the retirement village, where he resumed limited baseline functional activity. The patient was reviewed in the outpatient surgical clinics three months postoperatively, and there was no recurrence of the hernia detected clinically on physical examination (Figure 5).

## Discussion

Giant inguinoscrotal hernias have become increasingly rare in developed countries due to earlier diagnosis, improved access to medical care, and timely surgical intervention. Today, they are more commonly encountered in rural populations or among individuals with self-neglect [1,4]. These hernias account for approximately 5% of all inguinal hernias, with bilateral involvement occurring in 12.5% of cases [5]. The management of giant inguinoscrotal hernias presents significant surgical challenges, not only due to the technical complexity of repair but also because affected patients often have multiple pre-existing comorbidities. Surgical trainees and junior consultants may have limited exposure to these cases, particularly in emergency settings, where preoperative optimization and careful patient selection may not be possible.

In some patients with severe comorbidities, general anesthesia may pose excessive risk. In the absence of significant symptoms or acute complications such as bowel strangulation, conservative management may be necessary. While pain and discomfort are often used to assess symptom severity, other quality-of-life issues must also be considered in these patients. These include difficulty voiding, urinary retention, and skin irritation due to persistent moisture and dribbling from a buried penis. Lymphatic and venous congestion in the scrotal skin increases the risk of excoriation, ulceration, and secondary infections [1,6]. Beyond physical symptoms, the socioeconomic and psychological consequences of giant inguinoscrotal hernias are often overlooked. Impaired mobility can restrict employment opportunities, while poor self-esteem and sexual dysfunction may significantly impact mental health and intimate relationships [7]. These factors further highlight the importance of a comprehensive, patient-centered approach to managing this condition.

One of the critical challenges in repairing giant inguinoscrotal hernias is loss of domain, a condition in which a significant portion of the abdominal viscera resides outside the abdominal cavity. When these herniated contents are forcibly reduced into a contracted peritoneal cavity, fascial closure may become difficult or even impossible. More importantly, this sudden increase in intra-abdominal volume can lead to elevated IAP, posing a risk of abdominal compartment syndrome (ACS) [6]. Elevated IAP can result in diaphragmatic dysfunction, ranging from mild atelectasis, which increases the risk of postoperative respiratory infections, to severe cases requiring postoperative mechanical ventilation. In critical scenarios, ACS can lead to reduced venous return, impaired cardiac output, and diminished microcirculatory blood flow, ultimately precipitating multiorgan dysfunction [8].

Currently, there are no well-defined surgical parameters to accurately predict the development of ACS following hernia repair. Furthermore, the clinical correlation between IAP measurements and patient outcomes remains unclear in the literature, and as such, IAP monitoring is not yet standard practice [9]. Despite this, surgeons must remain vigilant for potential postoperative complications. Close monitoring of vital signs and urine output is essential, and a high index of suspicion should be maintained for early detection of ACS [10]. Prompt recognition and intervention are crucial in mitigating the life-threatening consequences associated with this condition. Various techniques, such as visceral debulking, progressive pneumoperitoneum (PPP), phrenectomy, and botulinum toxin injections to the abdominal wall, have been previously utilized to mitigate the effects of loss of domain [1]. However, these strategies are typically reserved for elective repairs. In emergency settings with suspected bowel compromise, visceral debulking is a viable option but introduces additional complexity, increasing operative time and the risk of contamination or anastomotic complications [11].

In addition to the challenges posed by loss of domain, the repair of large bilateral anatomical defects further complicates surgical management. This case report describes the application of the Rives-Stoppa repair, a technique that evolved from the original Stoppa repair described in 1965. The Stoppa repair, also known as GPRVS, involves placing a large mesh in the preperitoneal space to provide comprehensive reinforcement of the lower visceral peritoneal sac. The technique relies on IAP to secure the mesh while the peritoneum acts as a barrier between the prosthesis and visceral contents. The subsequent evolution of this approach into the Rives-Stoppa repair expanded its application to complex ventral hernias. Above the arcuate line, mesh is placed in the retrorectus plane, with the posterior rectus sheath providing dorsal separation from visceral contents [3].

This clinical scenario presented an ideal opportunity for the application of the Rives-Stoppa repair, as the abdominal cavity was accessed for a right hemicolectomy, predisposing the patient to future midline ventral hernia development. This technique was preferred due to its suitability in addressing extensive and chronic hernia defects. When utilizing the Rives-Stoppa approach, recurrence rates are relatively low, at 1.9% for groin hernias and [12] 8% for ventral incisional hernias [13]. Alternative repair techniques for inguinal hernias include the open anterior approach, which has been successfully described in many case reports of giant inguinoscrotal hernias; however, long-term recurrence rates remain poorly defined. Traditional open anterior repair techniques - such as Bassini or Lichtenstein repairs using 12 cm or 15 cm mesh - fail to provide the necessary 5 cm overlap for large hernia defects. This inadequate coverage is particularly problematic in giant inguinoscrotal hernias, contributing to persistent weaknesses and a high risk of early recurrence [14]. Other alternatives include laparoscopic transabdominal preperitoneal (TAPP) repair and laparoscopic totally extraperitoneal (TEP) repair. However, laparoscopic approaches are often unfeasible in emergency cases with dilated bowel loops, significant loss of domain, and an inability to reduce hernia contents. In such complex cases, open posterior approaches such as the Rives-Stoppa repair remain a reliable and durable solution, particularly when addressing extensive hernia defects in patients with altered abdominal wall dynamics [4].

## Conclusions

In conclusion, giant bilateral inguinoscrotal hernias pose significant surgical challenges, especially in emergency settings. This case underscores the importance of careful risk assessment, operative planning, and vigilant postoperative care. The Rives-Stoppa repair provides a durable solution for extensive defects, minimizing recurrence and restoring the abdominal domain. Greater awareness and training are essential to improve outcomes in these rare and complex cases.
